# A salivary metabolite signature that reflects gingival host-microbe interactions: instability predicts gingivitis susceptibility

**DOI:** 10.1038/s41598-020-59988-z

**Published:** 2020-02-20

**Authors:** Marcela M. Fernandez-Gutierrez, Sultan Imangaliyev, Andrei Prodan, Bruno G. Loos, Bart J. F. Keijser, Michiel Kleerebezem

**Affiliations:** 1 TI Food and Nutrition, Nieuwe Kanaal 9-A, 6709 PA Wageningen, The Netherlands; 2 Host-Microbe Interactomics Group, Department of Animal Sciences, Wageningen University & Research, De Elst 1, 6708 WD Wageningen, The Netherlands; 3 TNO Microbiology and Systems Biology, Utrechtseweg 48, 3704 HE Zeist, The Netherlands; 4 Department of Oral Biochemistry, Academic Centre for Dentistry Amsterdam (ACTA), University of Amsterdam and Vrije Universiteit Amsterdam, Gustav Mahlerlaan 3004, 1081 LA Amsterdam, The Netherlands; 5 Department of Periodontology Academic Centre for Dentistry Amsterdam (ACTA), University of Amsterdam and Vrije Universiteit Amsterdam, Gustav Mahlerlaan 3004, 1081 LA Amsterdam, The Netherlands; 6 Department of Preventive Dentistry, Academic Centre for Dentistry Amsterdam (ACTA), University of Amsterdam and Vrije Universiteit Amsterdam, Gustav Mahlerlaan 3004, 1081 LA Amsterdam, The Netherlands

**Keywords:** Biochemistry, Risk factors

## Abstract

Several proteins and peptides in saliva were shown to stimulate gingival wound repair, but the role of salivary metabolites in this process remains unexplored. *In vitro* gingival re-epithelialization kinetics were determined using unstimulated saliva samples from healthy individuals collected during an experimental gingivitis study. Elastic net regression with stability selection identified a specific metabolite signature in a training dataset that was associated with the observed re-epithelialization kinetics and enabled its prediction for all saliva samples obtained in the clinical study. This signature encompassed ten metabolites, including plasmalogens, diacylglycerol and amino acid derivatives, which reflect enhanced host-microbe interactions. This association is in agreement with the positive correlation of the metabolite signature with the individual’s gingival bleeding index. Remarkably, intra-individual signature-variation over time was associated with elevated risk for gingivitis development. Unravelling how these metabolites stimulate wound repair could provide novel avenues towards therapeutic approaches in patients with impaired wound healing capacity.

## Introduction

Oral wounds can result from daily activities (e.g. tooth brushing, chewing and eating), but also from surgery, trauma or presence of inflammatory conditions such as periodontal diseases. Unlike skin injuries, wounds in the oral cavity are characterized by a higher repair rate and lack of scar formation^1^. The rapid wound repair observed in the oral cavity has been attributed to several factors. First, oral epithelial cells have a higher turnover rate than skin cells allowing a faster repair of the oral mucosa^2^. Second, there is a better microcirculation in the oral mucosa than in the skin, facilitating faster recruitment of immune cells to the site of injury that can effectively remove phagocytized bacteria and cell debris^3^. Third, the presence of saliva provides a humid environment that promotes re-epithelialization through a faster cell migration^4^. In addition, the role of individual salivary proteins or peptides on maintenance of oral health has been extensively studied^5^. For example, epidermal growth factor (EGF)^6^ and transforming growth factor alpha (TGFα)^7^ play an important role during oral wound repair through the activation of several signalling cascades that lead to migration, differentiation, and proliferation of cells. Similarly, vascular endothelial growth factor (VEGF) is involved in re-epithelialization, extracellular matrix regulation and angiogenesis, which are all critical processes that take place during wound repair^8^. Certain antimicrobial peptides present in saliva such as β-defensins and cathelicidin have also been reported to have growth stimulating properties in keratinocytes and lung cells^9,10^. In particular, histatin (Hst) 1 and Hst 2, primarily recognized for their antimicrobial activity in the oral cavity, have been identified as potent stimulators of cell migration via activation of the ERK 1/2 pathway in oral cells^11^. At the same time, saliva is also a rich source of metabolites or small molecules (<2,000 Da), which unlike the salivary proteome, have not been characterized in detail. Recently, a total 853 human salivary metabolites were identified and annotated using a multi-platform approach in combination with computer aided literature-mining^12^. This metabolite collection complemented the work of the Human Metabolome Database (HMDB) (http://www.hmdb.ca/) which provides the most up-to-date and comprehensive library of the human metabolome^13^.

In a previous study, we examined the changes in the salivary metabolome of 61 healthy adults at 5 timepoints during an experimental gingivitis challenge intervention^14^. The intervention required the volunteers to refrain from any form of oral hygiene for two weeks, resulting in plaque accumulation and induction of mild inflammation (i.e. gingivitis). Using untargeted metabolomics, we measured and identified 497 metabolites in a total of 305 unstimulated saliva samples collected during the challenge period of the study^14^. Here we identified a metabolite signature composed of ten compounds of which the relative concentrations are able to predict the effect of a saliva sample on gingival re-epithelialization kinetics using a recently developed *in vitro* model^15^. Several of the compounds of the signature are associated with increased cell damage and inflammation possible due to increased host-microbe interactions. This was reflected by a positive association between the predicted gingival re-epithelialization capacity elicited by the saliva samples and the inflammatory state of the periodontal tissues at the moment of saliva sampling. Finally, instability over time of the salivary metabolite signature was correlated with an increased response to the experimental gingivitis challenge.

## Results

### Stimulatory capacity of saliva on re-epithelialization is highly variable among individuals and potentially influenced by salivary metabolites

**Figure 1:**
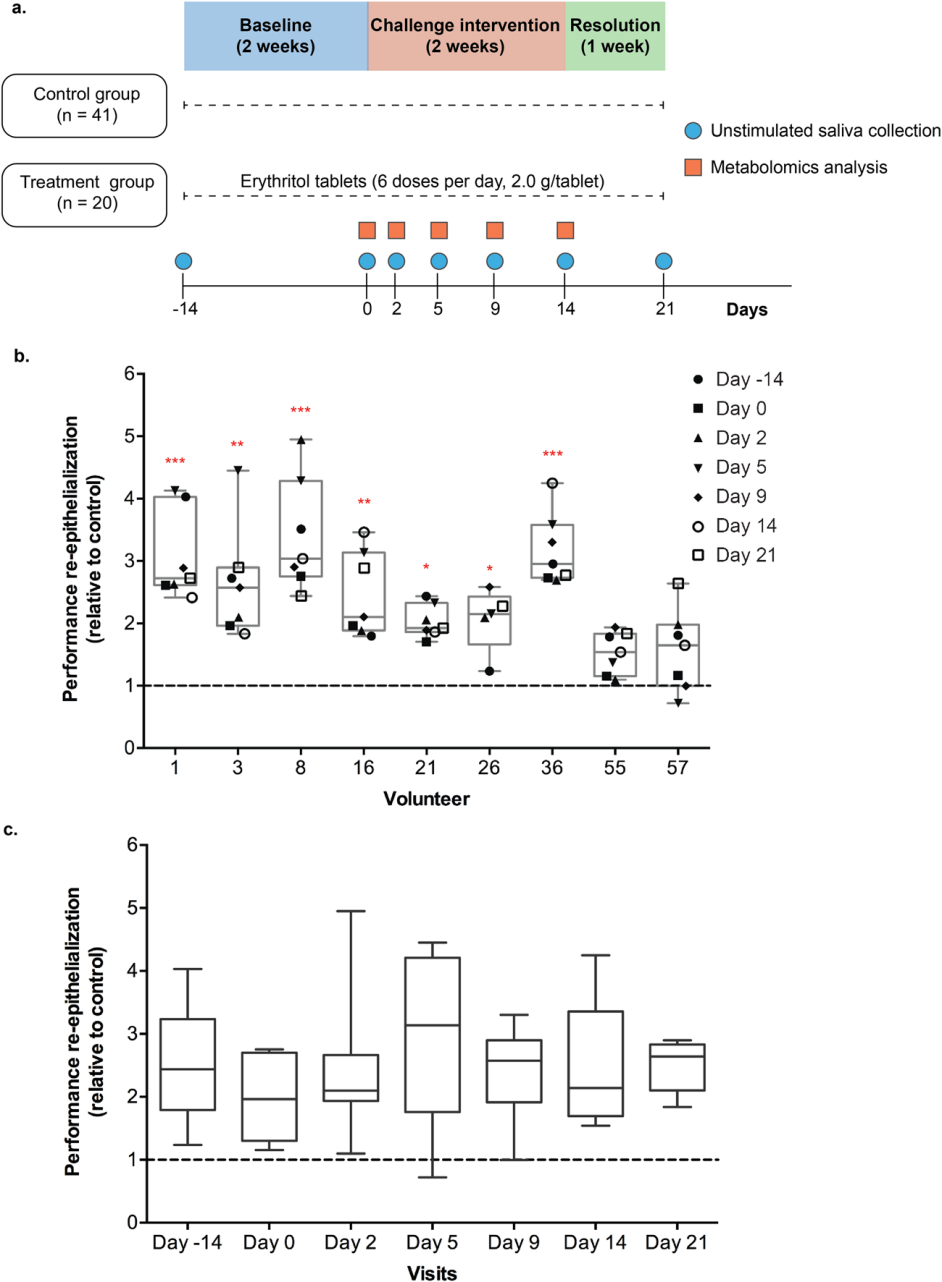
Study design and saliva effect on re-epithelialization kinetics. (**a**) Design of the challenge intervention, randomized study, adapted from Prodan *et al*.^14^. (**b**) Effect of the selected unstimulated saliva on re-epithelialization kinetics. Volunteers 1, 3, 16, 21, 55 and 57 corresponded to the control group. Volunteers 8, 26 and 36 corresponded to the treatment group. Dashed line represents the non-treated control. (**c**) Re-epithelialization capacity of unstimulated saliva collected at different timepoints of the challenge intervention study. Significant differences from the non-treated control were assessed by a one-way ANOVA followed by a Dunnett’s test for multiple comparisons (*P < 0.05; **P < 0.001; ***P < 0.0001).

To investigate if changes in the salivary metabolome during the development of gingivitis could alter the salivary capacity to induce wound repair, we evaluated the effect of unstimulated saliva samples collected at the baseline, challenge intervention, and resolution phases of a previous study^14^ (also see study design in Methods section and Fig. 1a) on the re-epithelialization kinetics of gingival epithelial cells (i.e. Ca9–22 cell line). Confluent cell monolayers were scratched and treated with unstimulated saliva samples obtained from 9 randomly selected participants at the 7 timepoints of the study (Day -14 to Day 21; Fig. 1b). Live-imaging followed by modelling of the re-epithelialization curves were conducted to obtain kinetic parameters that quantitatively describe wound repair (μ_m_ and A parameters; see^15,16^ and Methods section for details). The overall impact of the treatment with specific saliva samples on wound-repair kinetics was assessed by the re-epithelialization performance value (μ_m_*A) that was expressed relative to the untreated control within the same assay run^15^. Even though the effect of unstimulated saliva on oral re-epithelialization kinetics was highly variable among individuals (Fig. 1b), the saliva samples derived from most individuals resulted in significantly enhanced re-epithelialization kinetics relative to the non-treated control. The stimulatory effect observed for saliva samples from specific individuals did not show a consistent change during the time-course of the study (Fig. 1b,c), indicating that the wound-repair capacity of saliva was not consistently influenced during the experimental gingivitis challenge. In addition, neither daily intake of erythritol or gender had a significant influence on the salivary capacity to promote wound repair (Fig. S1), and no significant association was found between total protein content and the capacity of the saliva samples to promote gingival re-epithelialization (Fig. S2).

### Metabolite signature in saliva is associated with re-epithelialization kinetics in oral cells

**Figure 2:**
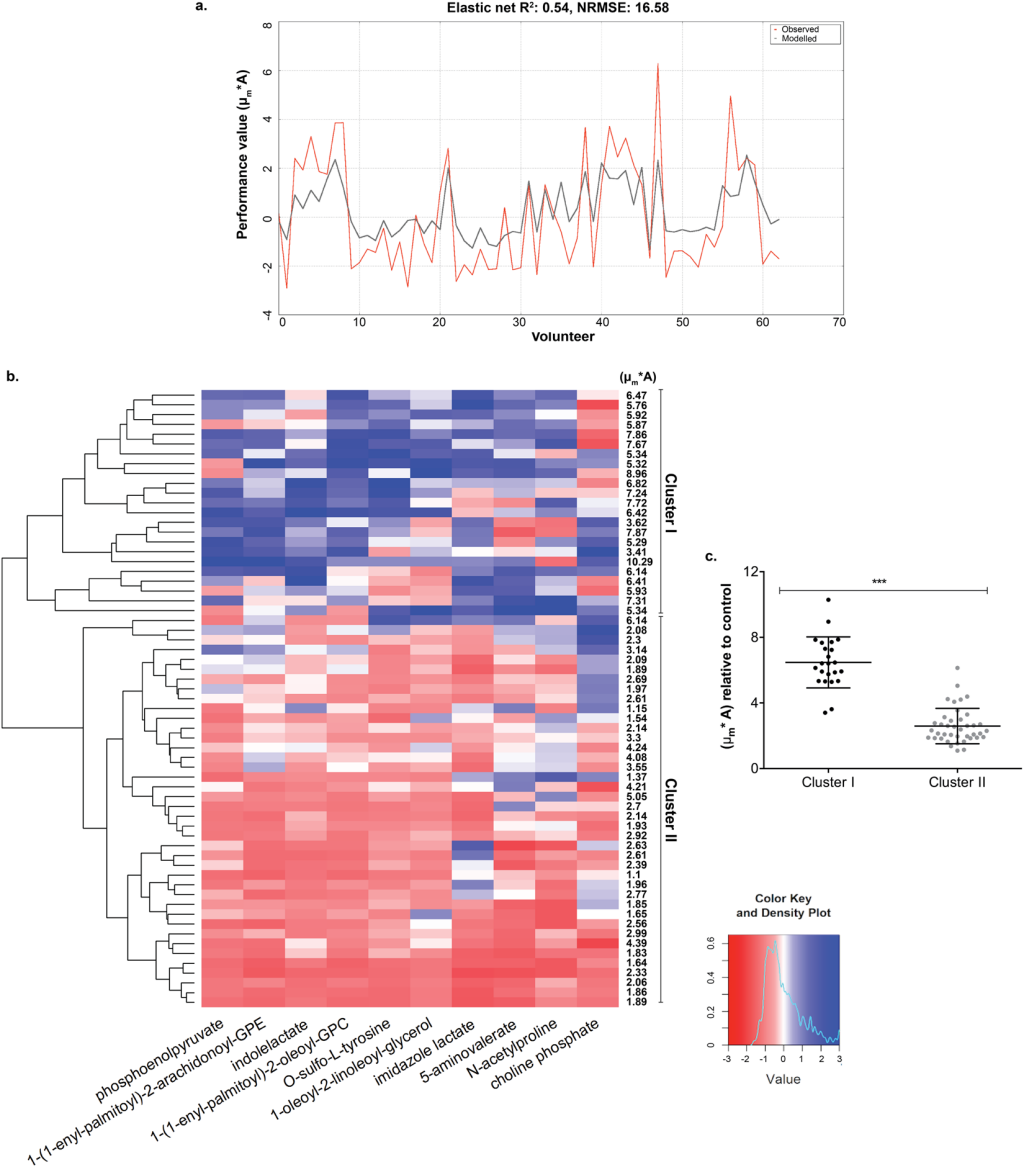
Salivary metabolite signature related to observed re-epithelialization kinetics. (**a**) Performance of the elastic net regression through the (μ_m_*A) parameter values. (**b**) Hierarchical clustering heatmap displaying relative metabolite concentrations of the signature and observed re-epithelialization kinetic values (μ_m_*A) for the training dataset. (**c**) Re-epithelialization performance induced by saliva samples of clusters I and II derived from the hierarchical clustering. Significant difference was assessed by a two-tailed t-test (***P < 0.0001).

To investigate whether the chemical composition of saliva influences the differential re-epithelialization capacity observed between samples, we performed elastic net regressions^17^ with stability selection^18^. To increase the power of this analysis, the kinetic parameters determined for the saliva samples collected during the challenge intervention period (Day 0 to Day 14, see Fig. 1a) was expanded with 20 randomly selected samples (Table S1; different individuals and different timepoints) for which metabolome data was available (i.e. excluding samples collected at Day -14 and 21). The filtered and scaled metabolite dataset (see Methods section for more details) together with each of the scaled re-epithelialization parameter values (i.e. μ_m_, A, and μ_m_*A) were employed in the elastic net regressions. The best fit of the model (R^2^ = 0.54) with the lowest normalized root mean-square-error^19^ (NRMSE = 16.58) when compared with the actual measurements were obtained with the performance values (μ_m_*A) (Fig. 2a). Therefore, these values were used as a training dataset to perform elastic net regression analysis in combination with stability selection^18^, leading to the identification of a set of metabolites that collectively were associated with the observed oral re-epithelialization (Table 1). Notably, the use of the μ_m_ parameter in the elastic net regression also resulted in a very good fit (R^2^ = 0.52, NRMSE = 20.19, see Fig. S3) and the selection of a partly overlapping metabolite set compared to the analysis using the performance values, which included the plasmalogen lipids and the O-sulfo-L-tyrosine (Table S2). This finding suggests that the μ_m_ parameter has a greater contribution to the model than the A parameter.

**Table 1:** Metabolite signature.

Metabolite name	Description	Stability	Average Weight
1-(1-enyl-palmitoyl)-2-arachidonoyl-GPE (P-16:0/20:4)	Plasmalogen	0.69	0.14
1-(1-enyl-palmitoyl)-2-oleoyl-GPC (P-16:0/18:1)	Plasmalogen	0.67	0.09
1-oleoyl-2-linoleoyl-glycerol (18:1/18:2)	Diacylglycerol	0.75	0.11
5-aminovalerate	Lysine metabolism	0.67	0.07
choline phosphate	Phospholipid metabolism	0.63	−0.17
imidazole lactate	Histidine metabolism	0.88	0.17
Indolelactate	Tryptophan metabolism	0.72	0.10
N-acetylproline	Urea cycle; arginine and proline metabolism	0.79	0.14
O-sulfo-L-tyrosine	Chemical	0.91	0.21
phosphoenolpyruvate (PEP)	Glycolysis, gluconeogenesis and pyruvate metabolism	0.73	0.14

Elastic net regression with stability selection was performed to select a set of metabolites that were associated to re-epithelialization kinetics (μ_m_*A).

All the metabolites in the identified signature had a high stability (>0.6), indicating that the metabolites were selected in more than 60% of the subsampling iterations (n = 100) performed. In particular, O-sulfo-L-tyrosine had a very high stability and was selected in 91% of the iterations. Due to the high stability of O-sulfo-L-tyrosine in the elastic net regression, we evaluated the potential of this single metabolite as a biomarker of saliva-induced re-epithelialization by performing a linear regression to model the relationship between the relative concentration of O-sulfo-L-tyrosine and the re-epithelialization kinetics measured in the *in vitro* assay. The fit of this single metabolite (R^2^ = 0.23, p-value = 4.21 × 10^−5^) was much lower than the fit obtained in a multiple linear regression that included all the metabolites in the identified signature (R^2^ = 0.74, p-value = 4.80 × 10^−14^). Moreover, analysis of variance between the two models showed that the addition of all the metabolites in the signature significantly contributed to the observed re-epithelialization kinetics (F = 2.01, p-value = 2 × 10^−11^). These observations strongly support the higher biomarker-value of the metabolite signature as compared to the best-performing single metabolite within the signature.

Besides choline phosphate, all other selected metabolites in the elastic net regression had a positive average weight, indicating that increasing concentrations of these metabolites were associated with enhanced re-epithelialization kinetics. A hierarchical clustering of the relative concentrations of the metabolites in the identified salivary signature revealed two main clusters in the saliva sample set (Fig. 2b). In agreement with the elastic net regression analysis, the majority of the saliva samples in the upper cluster (I) contained relatively high concentrations of the signature metabolites that led to high re-epithelialization kinetics in gingival cells. Only for choline phosphate, the relation between its relative concentration and the re-epithelialization kinetics was inversed. Conversely, the lower cluster (II) grouped saliva samples with relative low concentration of most signature metabolites and induced lower re-epithelialization kinetics. This cluster differentiation is further substantiated by the significant difference in re-epithelialization kinetics between the sample groups (p-value < 0.0001) (Fig. 2c). Notably, hierarchical clustering of randomly selected metabolites from the dataset consistently failed to separate the saliva samples into two distinctive clusters with distinct re-epithelialization kinetics (a total of 70 heatmaps; see Fig. S4 for two random examples), underpinning the specificity of the metabolite signature identified.

### Prediction and validation of oral re-epithelialization kinetics using the salivary metabolite signature

**Figure 3:**
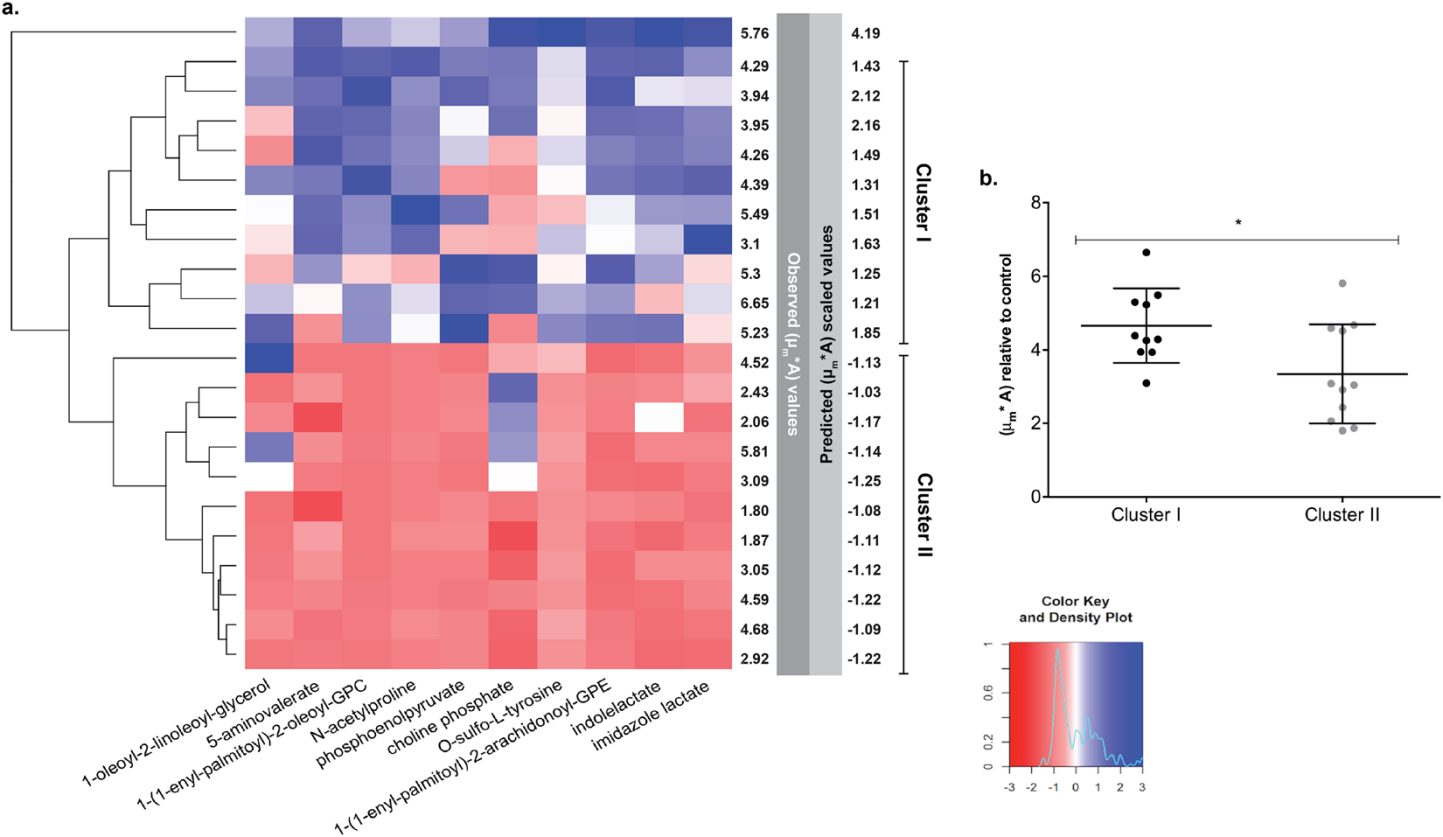
Predictive capacity of the salivary metabolite signature. (**a**) Hierarchical clustering heatmap displaying relative metabolite concentrations of the signature and re-epithelialization performance for the validation dataset. (**b**) Re-epithelialization performance observed in clusters I and II. Significant difference was assessed by a two-tailed t-test (*P < 0.05).

To validate the predictive potential of the metabolite signature, we used it to predict the re-epithelialization capacity of the remaining 242 unstimulated saliva samples collected during the challenge intervention period of the study (n = 305) (Fig. 1a). Subsequently, we selected previously untested saliva samples that were predicted to have among the highest and lowest stimulatory capacity (n = 11 in each group) in the re-epithelialization assay. The re-epithelialization kinetics of gingival cells stimulated with these samples was determined and showed that the metabolite signature could successfully predict the relative re-epithelialization capacity of the saliva samples (Fig. 3). Similar to the training dataset, the metabolite signature separated the samples in two distinct clusters (I and II; Fig. 3a) that induced significantly higher (cluster I) and lower (cluster II) wound repair kinetics (p-value = 0.02) (Fig. 3b). Moreover, Spearman correlation analysis corroborated a significant association between the observed and predicted kinetics of re-epithelialization (r = 0.44, p-value = 0.043, n = 22). Taken together, the combination of several metabolites in the signature presented in this study has a powerful predictive potential of the effect of the saliva in oral re-epithelialization.

### Relevance of the metabolite signature in relation to gingival bleeding and the magnitude of the response to the challenge

**Figure 4:**
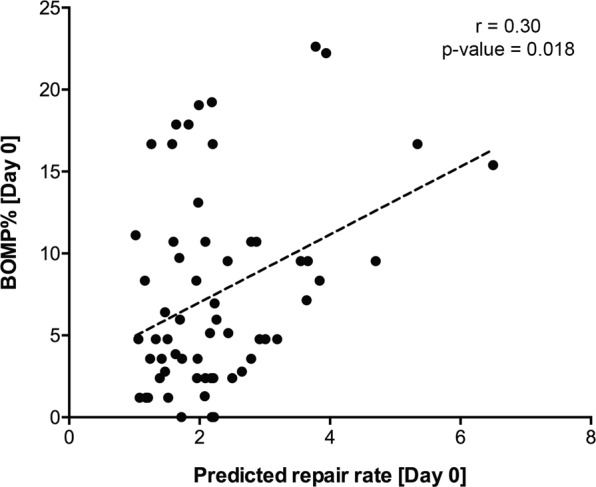
Saliva re-epithelialization capacity in relation to gingival bleeding. Spearman correlation analysis between the predicted saliva re-epithelialization capacity and the percentage of gingival bleeding in 61 individuals at the baseline (Day 0) of the study.

Periodontal diseases are multifactorial inflammatory diseases that lead to damage of the periodontal tissues supporting the teeth^20^. The gingival bleeding index^21^ has long been used as a reliable clinical parameter for evaluating the inflammatory conditions of the periodontium and over time has been modified in several ways, for example, by scoring for the absence or presence of gingival bleeding on marginal probing (BOMP)^22^. To evaluate if there was an association between the re-epithelialization capacity predicted by the metabolite signature and this clinical parameter (percentage of gingival bleeding), we conducted Spearman correlation analysis at the baseline of the study (Day 0). This analysis revealed a significant positive association between these two variables with a correlation coefficient of 0.30 (p-value = 0.018) (Fig. 4). This suggests that volunteers whose saliva samples displayed a higher stimulatory effect on re-epithelialization had increased gingival inflammation in comparison to the individuals whose saliva samples elicited lower stimulatory capacity. Next, we evaluated the predictive capacity of the metabolite signature at baseline in relation with the response to the challenge which was reflected by the change in gingival bleeding between the peak and baseline of the challenge (Δ BOMP%). Individuals were median split into two groups: (1) those whose samples were predicted to have higher re-epithelialization capacity and (2) those with lower capacity. No significant difference was found between the two groups (Fig. S5), suggesting that the metabolite signature has no predictive capacity with respect to the magnitude of the response to the challenge.

**Figure 5:**
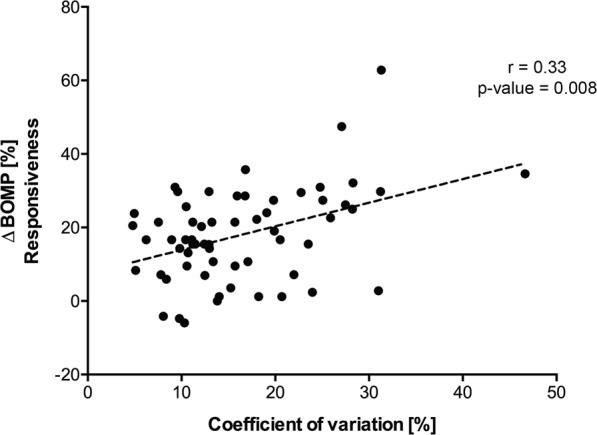
Effect of intra-individual variation in response to the experimental gingivitis challenge. Spearman correlation analysis between the coefficient of variation (%), obtained with the predicted re-epithelialization values calculated during the challenge period (Day 0, 2, 5, 9, and 14), and change in gingival bleeding between the baseline and the peak of the challenge (ΔBOMP%) in 61 individuals.

Nevertheless, we set out to investigate if intra-individual variation of the predicted re-epithelialization capacity of the saliva samples collected at different timepoints could influence the magnitude of the response to the challenge. For this, we calculated the coefficient of variation^23^, which describes the variability of the predicted repair values relative to the mean re-epithelialization capacity of the samples collected for each of the volunteers in the study (n = 61). Spearman correlation analysis revealed a significant positive association (r = 0.33, p-value = 0.008) between the coefficient of variation of the predicted re-epithelialization capacity and the change in gingival bleeding during the trial (Δ BOMP%) (Fig. 5), suggesting that the individuals with higher variability in their metabolite signature display an increased response to the accumulation of plaque during the experimental gingivitis challenge and potentially have a higher risk to develop gingivitis.

## Discussion

Human saliva is secreted from three pairs of major salivary glands (parotid, submandibular, and sublingual glands) as well as numerous minor salivary glands lying beneath the oral mucosa^24^. The protective role of saliva in the maintenance of oral health can be attributed to the presence of its inorganic and organic constituents. Among the organic constituents, saliva contains a myriad of proteins and peptides that have been shown to modulate different aspects of oral health, including enzymatic digestion, food tasting and swallowing, lubrication, protection against pathogenic microorganisms, remineralization, and stimulation of tissue repair^3,5,24–26^.

In recent years, metabolomic analysis of human saliva have revealed that this ‘biofluid’ is also a rich source of low molecular weight metabolites such as lipids, amino acids, vitamins, organic acids, thiols, carbohydrates, and their derivatives, which can be important indicators of physiological or pathological states^12,27,28^. Different studies have shown that specific changes in the metabolic profiles of saliva can be associated with the presence of periodontal diseases^29^ and oral cancer^30^. Importantly, metabolites from blood can pass into the saliva through transcellular, intracellular or paracellular routes involving passive diffusion or active transport within the salivary glands or the gingival sulcus^31^. As a consequence salivary metabolites have the potential to provide a window to the rest of the body that has been used in the early detection of bacterial infections^32^, breast cancer, and pancreatic cancer^30^.

In this study we used the metabolite profiles of 305 unstimulated saliva samples collected from 61 healthy volunteers at 5 timepoints during an experimental gingivitis challenge intervention^14^ to investigate their influence on re-epithelialization kinetics using a quantitative *in vitro* scratch model^15^. The re-epithelialization kinetics (in particular the overall performance value μ_m_*A) obtained in a training set of saliva samples (n = 63) enabled the identification of a specific salivary metabolite signature (10 metabolites) that allowed the prediction (and partial verification) of the re-epithelialization stimulatory capacity of saliva samples obtained in an experimental gingivitis challenge model. Importantly, regression analyses revealed that the metabolite signature correlated much stronger with the observed re-epithelialization kinetics than single metabolites within the signature, which clearly illustrates the benefit of combinatorial multi-factorial biomarkers as compared to single factor biomarkers. Similarly, in a recent proteomics study, the authors identified a panel of clinical biomarkers in saliva that reflect the transition between health and periodontal disease and could serve as a better diagnostic tool as opposed to the classical single biomarker evaluation^33^.

The metabolite signature (Table 1) contained several metabolites that are associated with host-microbe interactions, cellular degradation, and in some cases inflammation. The 1-(1-enyl-palmitoyl)-2-arachidonyl-GPE and 1-(1-enyl-palmitoyl)-2-oleoyl-GPC are plasmalogens, a type of membrane phospholipids that contain a vinyl-ether bond at the *sn-*1 position of the glycerophospholipid backbone^34^. Plasmalogens comprise ~18% of the phospholipid mass in humans and help maintaining the physical bilayer properties of cell membranes^35^, while the hydrogen atoms adjacent to the vinyl-ether bond are preferentially oxidized when exposed to free radicals and thereby can act as antioxidants^36,37^. Untargeted metabolic profiling of saliva from healthy and periodontal patients, revealed an association between periodontal disease and elevated levels of degradation products of several macromolecules, including glycerolphospholipids^29^. Moreover, elevated concentrations of various plasmalogen derivates in serum and/or synovial fluid have been correlated with the severity of inflammatory diseases like obesity-associated osteoarthritis (OA) and rheumatoid arthritis in both human^38^ and mice^39^. Interestingly, the metabolite 1-oleoyl-2-linoeoyl-glycerol (diacylglycerol; DAG) has recently been associated with cell damage and the formation of spatially organized lipid domains around the site of injury^40^, in which DAG was shown to be required for regulation of GTPases, Rho and Cdc42 involved in cortical cytoskeleton repair^40^. Choline phosphate (ChoP) is an intermediate in the synthesis of phosphatidylcholine, a major phospholipid of eukaryotic cell membranes^41^. ChoP has been shown to potentiate the mitogenic effect of insulin in NIH 3T3 fibroblasts^42,43^, and therefore it remains unclear why increasing concentrations of this metabolite in saliva resulted in reduced re-epithelialization capacity. Taken together, higher concentrations of these lipid metabolites (with the exception of choline phosphate) in saliva may result from increased mucosal damage and cellular decay through enhanced host-microbe interactions, as was already proposed by the authors of the salivary metabolome study^29^. Moreover, such elevated host-microbe interplay is in line with the higher levels of modified amino acids that (potentially) result from bacterial metabolism, i.e. 5-aminovalerate, imidazole lactate, indolelactate, N-acetylproline, and O-sulfo-L-tyrosine. In particular, elevated levels of 5-aminovalerate, a metabolite of the foul-smelling diamine cadaverine that is produced by microbial protein hydrolysis^44^, has been associated with patients with chronic periodontitis^45^. Finally, many secreted and transmembrane proteins that play a role in various biological processes involved in wound repair (e.g. blood coagulation, optimal rolling of leukocytes on endothelial cells upon tissue damage, chemokine receptor signalling, and extracellular matrix remodelling) are commonly post-translationally modified when they pass through the trans-golgi compartment to introduce O-sulfo-tyrosine residues^46–48^. Hence, the elevated levels of this metabolite in saliva may reflect increased bacterial hydrolysis of host-derived proteins.

Overall, the metabolite signature identified in this study encompasses metabolites that could reflect enhanced host-microbe interactions at the mucosal tissues in the oral cavity and may thus, reflect a chemical signature for mild mucosal damage. Higher concentrations of most of the metabolites in the signature (with the exception of choline phosphate) was associated with accelerated gingival wound repair *in vitro*, which could reflect the cellular response to the proposed mucosal damage, and which is in agreement with the signature’s association with elevated gingival bleeding scores. These results seem to support earlier findings on the proteomic profile of the secretome of gingival epithelium in response to biofilm formation in which deregulation of host responses involved in cytoskeletal rearrangement, stress responses and epithelial tissue integrity, among others were observed^49,50^.

Intriguingly, we also found a significant positive association between the intra-personal variation of the metabolite signature and the magnitude of the response to the challenge, reflected by increased gingival bleeding scores during the experimental gingivitis trial. One may speculate that stability of host-microbe interactions in the oral cavity, and its corresponding salivary metabolite signature, could reflect a healthy state. In such context, individuals with unstable host-microbe interactions and higher variability of the metabolite signature may be at higher risk to respond more strongly under a challenge, like the gingivitis challenge model tested in this study.

The mechanism by which the combination of these metabolites is able to modulate oral re-epithelialization remains unclear at this stage. They may directly, or indirectly activate wound-repair responses in gingival cells, which is reflected by enhanced re-epithelialization in the *in vitro* scratch assay, and this model may serve as an attractive assay system to unravel the mechanism of action of the metabolite signature. Finally, the identified signature may open promising avenues for the development of novel treatment strategies in patients with mucositis, recurrent oral aphthous ulcers or Sjögren’s syndrome, disease states that are all characterized by impaired wound healing^51,52^.

## Methods

### Study population and design

A challenge intervention, randomized, blinded study was carried out in the Academic Centre for Dentistry Amsterdam (ACTA) within the framework of the Top Institute of Food and Nutrition (TIFN) project “An exploratory study on the dynamic (microbial, biochemical and immunological) interactions of the oral ecosystem during induction of mild gingival inflammation”. The study population consisted of 61 systemically healthy adults (21 males, 40 females), between 18 and 55 years of age, with no apparent periodontitis or dental caries. Inclusion and exclusion criteria were previously described by Prodan *et al*.^14^. The study consisted of three phases: baseline, challenge intervention, and resolution phase (see Fig. 1a). The baseline phase consisted of two weeks (Day -14 to Day 0) in which the volunteers of the treatment group (n = 20) were requested to start taking six doses of erythritol per day (2 g/tablet) for the entire duration of the study (5 weeks), while the remaining participants (n = 41) were requested to follow their normal routine. Erythritol is a non-caloric polyol that is used as sugar substitute and has been shown to reduce plaque accumulation, limit growth of *Streptococcus mutans*, and lower caries rates *in vivo*^53–55^. The challenge intervention was based on the experimental gingivitis protocol developed by Löe *et al*.^56^ and encompassed a two-week period (Day 0 to Day 14) in which all participants (n = 61) were asked to refrain from any form of oral hygiene (i.e. tooth brushing, flossing, chewing gum, mouth wash), resulting in plaque accumulation and induction of mild gingival inflammation. This was followed by a resolution phase of one week (Day 14–Day 21) in which the volunteers reinitiated standardized oral hygiene practices to restore normal oral homeostasis.

### Ethical considerations

The study protocol was reviewed and approved by the Medical Ethics Committee of the Vrije Universiteit Medical Centre (2014_505) and registered at the Central Committee on Research Involving Human Subjects (CCMO). The study was conducted according to the Declaration of Helsinki^57^ of the World Medical Association. All volunteers signed an informed consent form.

### Unstimulated saliva collection

Unstimulated saliva samples were collected at seven timepoints across the study (Day -14, Day 0, Day 2, Day 5, Day 9, Day 14 and Day 21). Sample collection occurred between 9.00 and 12.00 AM. Volunteers were instructed not to eat or drink two hours prior to the appointment. For sample collection, volunteers were told to accumulate saliva on the floor of their mouth, without facial or tongue movements, and spit every 30 seconds for 5 minutes into a sterile polypropylene tube, which was kept on ice. Samples were centrifuged (10,000 × g, 10 minutes at 4 °C) to remove epithelial cell debris, bacteria, and food residues, and then stored at −80 °C. Total protein content was determined as described in^58^ using a Pierce BCA Protein Assay Kit (Thermo Fisher Scientific, MA, USA).

### Metabolomics analysis

Untargeted metabolite profiling of the saliva samples was outsourced to Metabolon® (Durham, NC, USA) and processed as described previously^14^. Briefly, metabolite extracts were divided into five aliquots: two for analysis by two separate reverse phase ultrahigh performance liquid chromatography-tandem mass spectroscopy (RP/UPLC-MS/MS) methods with positive ion mode electrospray ionization (ESI), one for analysis by RP/UPLC-MS/MS with negative ion mode ESI, one for analysis by hydrophilic-interaction chromatography (HILIC)/UPLC-MS/MS with negative ion mode ESI, and one sample was reserved as backup. All methods used an ACQUITY UPLC System (Waters Corporation, MA, USA) and a Q-Exactive™ high-resolution, accurate-mass spectrometer (Thermo Fisher Scientific) interfaced with a heated electrospray ionization (HESI-II) source and Orbitrap mass analyzer operated at 35,000 mass resolution. Extraction of raw data, quality control processing and identification of peaks were performed using Metabolon’s hardware and software. Identification of peaks was carried out by comparison with the Metabolon’s reference library, which contains the retention time/index (RI), mass to charge ratio (m/z), and chromatographic data on all molecules present in the library. Inter-day instrument tuning differences were corrected for each compound by registering the medians equal to one in every run and normalizing each data point proportionally. Normalized peak areas were scaled by the median value of each compound. Metabolome analyses were performed on the five timepoints (Day 0, Day 2, Day 5, Day 9 and Day 14) corresponding to the challenge intervention phase of the study (see also Fig. 1a). The complete dataset can be found in Prodan *et al*. (Supplementary Material 1)^14^.

### Assessment of bleeding on marginal probing (BOMP)

Assessment of bleeding on marginal probing (BOMP) was performed as previously described by Oliveira *et al*.^22^ at the baseline of the study (Day -14 and Day 0), at the peak of the challenge intervention (Day 14), and after the resolution period (Day 21). The teeth with exception of third molars were clinically examined at six sites per tooth (disto-vestibular, vestibular, mesio-vestibular, disto-lingual, lingual and mesio-lingual) in two contralateral quadrants (i.e. one upper and one lower). The number of gingival units that bled upon marginal probing within 30 seconds was recorded with scores from 0 to 2 (0 = no bleeding, 1 = pinprick, 2 = excessive bleeding). Gingival bleeding was expressed as a percentage of sites that bled upon probing (Table S3). The response to the challenge was determined by the Δ BOMP%, which was obtained by calculating the difference between the percentage of gingival bleeding at the end (Day 14) and at the beginning (Day 0) of the challenge.

### Cell line maintenance

The human epithelial gingival cell line, Ca9–22 (JCRB0625), was purchased from the National Institute of Biomedical Innovation JCRB Cell Bank (Osaka, Japan). Ca9–22 cells were routinely cultured at 37 °C in a humidified atmosphere containing 5% CO_2_, in Dulbecco’s Modified Eagle Medium (DMEM) containing Glutamax (Invitrogen, Paisley, UK) and supplemented with 10% fetal calf serum (FCS), 100 U/mL penicillin and 100 μg/mL streptomycin. The cells were passaged every three to four days_._

### Scratch assay and re-epithelialization kinetics modelling

Scratch assays and modelling of re-epithelialization kinetics were performed as previously described^15^. A detailed protocol is available in Protocol Exchange^16^. Briefly, cells were seeded in 96-well plates (BD Falcon™, Corning, NY, USA) at a density of 3.5 × 10^4^ cells/well and incubated overnight. Confluent cell monolayers were starved in FCS-free DMEM for 2 hours during which the cytoplasm and nuclei were fluorescently labelled with 2 μM CellTracker™ Red CMTPX (Molecular Probes, OR, USA) and 2 μg/ml Hoechst 33342 (Molecular Probes), respectively. After incubation, the HTSScratcher (Peira, Antwerpen, BE) was used to introduce uniform longitudinal scratches in the cell monolayers of the multi-well plate, and wells were washed twice with phosphate-buffered saline (PBS) to remove cell debris followed by immediate addition of the treatments in FCS-free DMEM. Unstimulated saliva samples were tested in duplicate in a 1:4 ratio (unstimulated saliva: FCS-free DMEM) and FCS-free DMEM was used as non-treated control. As positive control of cell migration and proliferation, we used 4 ng/ml human transforming growth factor alpha (TGFα; R&D Systems, MN, USA). A combination of p38 (SB203580; Cell Signaling Technology, MA, USA) and MEK1/2 (U0126, Cell Signaling Technology) inhibitors served as negative control at a concentration of 10 μM each. Bright-field and fluorescent images (577 nm and 350 nm) of the same frame of each well were acquired every 20 minutes during a period of 5 hours with the BD Pathway 855 Bioimaging System (BD Biosciences, CA, USA) using a 4x objective under controlled conditions (37 °C and 5% CO_2_). Image segmentation and feature extraction was performed using CellProfiler (version 2.1.1.) and FCS Express 4 Plus (De Novo Software, CA, USA) was used to record the number of cells infiltrating into the scratched area over time. The enumeration of infiltrating cells was modelled using the modified Gompertz function^59^ to extract three kinetic wound-repair parameters (μ_m,_ A, and λ). The μ_m_ parameter represents the repair rate (cells minute^−1^) and the A parameter represents the maximum number of cells inside the scratched area at the plateau phase of the repair curve. Previous studies have shown that the third parameter obtained by kinetic modelling (λ, representing the point of infliction or lag-phase) has limited biological relevance when using the Ca9–22 gingival cells as the migration process in this particular cell line starts almost instantly after the scratch is introduced^15^. Treatments of which the modelled wound-repair curve showed a goodness of fit (R^2^) lower than 0.9 were discarded from further analysis. Notably, a fully integrated pipeline for image segmentation, enumeration of cells inside and outside the scratched area, kinetic modelling and parameter extraction is available online through the Galaxy platform^16,60^. Finally, the overall wound repair-performance was assessed by the (μ_m_*A) value^15^ that is expressed relative to the non-treated control (FCS-free DMEM).

### Elastic net regression and stability selection method

Elastic net regression^17^ with stability selection^18^ was performed to select a set of features (i.e. metabolites) that were related to re-epithelialization kinetics. The elastic net regression with stability selection was implemented in Python using the scikit-learn package v.0.16.1^61^. The elastic net balancing parameter was set at 0.3 to 0.8 in 20 steps, whereas the alpha factor parameter ranged from 10^−2^ to 10^+^^2^ in 150 steps. A total of 63 unstimulated saliva samples collected from 19 volunteers during the challenge phase (Day 0 to Day 14) of an earlier experimental gingivitis study^14^ were tested in duplicates in the re-epithelialization model to obtain the kinetic parameters (μ_m_, A, and μ_m_* A) that were used to train the model (Table S1). The features were selected based on their stability after 100 runs of feature selection modelling on random data shuffles using the stability selection procedure. Model hyperparameters were estimated using an exhaustive grid search within a 5-fold cross-validation procedure on 80% of the training dataset and the model’s generalization error was assessed on the remaining 20% test dataset. The performance measure used for a regression task is a Normalized Root Mean Square Error (NRMSE). Missing metabolite values were assumed to be below the detection limits and were imputed with the minimum detected value for that metabolite across all samples. Metabolites with more than 25% missing measurements were removed from the dataset. The filtered metabolite measurements were scaled by removing the mean value of each metabolite (zero-mean) and then divided by the standard deviation. Re-epithelialization parameter values were zero-mean scaled. The selected features were used to predict the *in vitro* re-epithelialization kinetics (μ_m_* A) of the remaining 242 unstimulated saliva samples collected during the challenge phase^14^ from a total of 61 individuals (Table S3).

### Calculation of the coefficient of variation of the predicted re-epithelialization values

Intra-individual variation of the predicted re-epithelization capacity of the saliva samples collected during the challenge period (Day 0, 2, 5, 9, and 14) was determined by calculating their coefficient of variation (CV)^23^, defined as the ratio of the standard deviation (σ) to the mean (μ).

$$CV=(\frac{\sigma }{\mu })\times 100$$

To avoid approximation of the coefficient of variation to infinity when mean values are close to zero, the predicted re-epithelialization values were unscaled by adding 1+ the absolute minimum value of the dataset to each predicted value (Table S3).

### Other statistical analyses

Statistical significance tests and correlation analyses were performed in GraphPad Prism version 6.0 (GraphPad Software, CA, USA). Linear and multiple linear regressions were performed in R. Heatmaps with hierarchical clustering were created in R using the heatmap.2 function of the gplots package version 3.0.1^62^.

## Supplementary information

Supplementary Information.

## References

[1] Szpaderska AM, Zuckerman JD, DiPietro LA (2003). Differential injury responses in oral mucosal and cutaneous wounds. J. Dent. Res..

[2] Squier, C. A. & Kremer, M. J. Biology of oral mucosa and esophagus. *J. Natl. Cancer Inst. Monographs*, 7–15 (2001). 10.1093/oxfordjournals.jncimonographs.a003443 11694559

[3] Brand HS, Veerman EC (2013). Saliva and wound healing. Chin. J. Dent. Res..

[4] Svensjo T, Pomahac B, Yao F, Slama J, Eriksson E (2000). Accelerated healing of full-thickness skin wounds in a wet environment. Plast. Reconstr. Surg..

[5] Amerongen AVN, Veerman ECI (2002). Saliva – the defender of the oral cavity. Oral Dis..

[6] Zelles T, Purushotham KR, Macauley SP, Oxford GE, Humphreys-Beher MG (1995). Concise review: Saliva and growth factors: The fountain of youth resides in us all. J. Dent. Res..

[7] Mogi M, Harada M, Inagaki H, Minami M, Kojima K (1995). Transforming growth factor-α in human submandibular gland and saliva. J. Immunoassay.

[8] Keswani SG (2013). Role of salivary vascular endothelial growth factor (VEGF) in palatal mucosal wound healing. Wound Repair Regen..

[9] Niyonsaba, F. *et al*. Antimicrobial peptides human beta-defensins stimulate epidermal keratinocyte migration, proliferation and production of proinflammatory cytokines and chemokines. *J. Invest. Dermatol*. **127**, 594–604 (2007). 10.1038/sj.jid.5700599 17068477

[10] von Haussen Judith, Koczulla Rembert, Shaykhiev Renat, Herr Christian, Pinkenburg Olaf, Reimer Dietlind, Wiewrodt Rainer, Biesterfeld Stefan, Aigner Achim, Czubayko Frank, Bals Robert (2008). The host defence peptide LL-37/hCAP-18 is a growth factor for lung cancer cells. Lung Cancer.

[11] Oudhoff MJ (2008). Histatins are the major wound-closure stimulating factors in human saliva as identified in a cell culture assay. FASEB J..

[12] Dame ZT (2015). The human saliva metabolome. Metabolomics.

[13] Wishart DS (2013). HMDB 3.0—the human metabolome database in 2013. Nucl. Acids Res..

[14] Prodan A (2016). Effect of experimental gingivitis induction and erythritol on the salivary metabolome and functional biochemistry of systemically healthy young adults. Metabolomics.

[15] Fernandez-Gutierrez MM (2017). S*treptococcus salivarius* MS-oral-D6 promotes gingival re-epithelialization *in vitro* through a secreted serine protease. Sci. Rep..

[16] Fernandez-Gutierrez, M. M. *et al*. High-throughput screening model to quantify re-epithelialization kinetics. *Protoc. Exch*. (2019).

[17] Zou H, Hastie T (2005). Regularization and variable selection via the elastic net. J. R. Stat. Soc. Series B Stat. Methodol..

[18] Meinshausen N, Bühlmann P (2010). Stability selection. J. R. Stat. Soc. Series B Stat. Methodol..

[19] Hyndman RJ, Koehler AB (2006). Another look at measures of forecast accuracy. Int. J. Forecast..

[20] Pihlstrom BL, Michalowicz BS, Johnson NW (2005). Periodontal diseases. Lancet.

[21] Löe H (1967). The gingival index, the plaque index and the retention index systems. J. Periodontol..

[22] Oliveira SC (2015). Correlations between two different methods to score bleeding and the relationship with plaque in systemically healthy young adults. J. Clin. Periodontol..

[23] Goodier J (2011). The Cambridge dictionary of statistics (4th edition). Ref. Rev..

[24] Dodds MWJ, Johnson DA, Yeh C-K (2005). Health benefits of saliva: A review. J. Dent..

[25] Milanowski M, Pomastowski P, Ligor T, Buszewski B (2017). Saliva–volatile biomarkers and profiles. Crit. Rev. Anal. Chem..

[26] Dawes C (2015). The functions of human saliva: A review sponsored by the world workshop on oral medicine VI. Arch. Oral Biol..

[27] Zhang A, Sun H, Wang X (2012). Saliva metabolomics opens door to biomarker discovery, disease diagnosis, and treatment. Appl. Biochem. Biotechnol..

[28] Arakaki AK, Skolnick J, McDonald JF (2008). Marker metabolites can be therapeutic targets as well. Nature.

[29] Barnes VM (2011). Metabolomics reveals elevated macromolecular degradation in periodontal disease. J. Dent. Res..

[30] Sugimoto M, Wong DT, Hirayama A, Soga T, Tomita M (2010). Capillary electrophoresis mass spectrometry-based saliva metabolomics identified oral, breast and pancreatic cancer-specific profiles. Metabolomics.

[31] Spielmann N, Wong DT (2011). Saliva: Diagnostics and therapeutic perspectives. Oral Dis..

[32] Kaufman E, Lamster IB (2002). The diagnostic applications of saliva— a review. Crit. Rev. Oral Biol. Med..

[33] Bostanci N (2018). Targeted proteomics guided by label-free quantitative proteome analysis in saliva reveal transition signatures from health to periodontal disease. Mol. Cell. Proteomics.

[34] Braverman NE, Moser AB (2012). Functions of plasmalogen lipids in health and disease. Biochim. Biophys. Acta.

[35] Nagan N, Zoeller RA (2001). Plasmalogens: Biosynthesis and functions. Prog. Lipid Res..

[36] Broniec A (2011). Interactions of plasmalogens and their diacyl analogs with singlet oxygen in selected model systems. Free Radic. Biol. Med..

[37] Sindelar PJ, Guan Z, Dallner G, Ernster L (1999). The protective role of plasmalogens in iron-induced lipid peroxidation. Free Radic. Biol. Med..

[38] Kosinska MK (2015). Articular joint lubricants during osteoarthritis and rheumatoid arthritis display altered levels and molecular species. PLoS One.

[39] Wu C-L, Kimmerling KA, Little D, Guilak F (2017). Serum and synovial fluid lipidomic profiles predict obesity-associated osteoarthritis, synovitis, and wound repair. Sci. Rep..

[40] Vaughan EM (2014). Lipid domain–dependent regulation of single-cell wound repair. Mol. Biol. Cell.

[41] Richter K (2016). Phosphocholine – an agonist of metabotropic but not of ionotropic functions of α9-containing nicotinic acetylcholine receptors. Sci. Rep..

[42] Kiss Z, Chung T (1996). Choline phosphate and phorbol ester potentiate the mitogenic effect of insulin by competitive mechanisms in nih 3t3 fibroblasts. Biochem. Biophys. Res. Commun..

[43] Tomono M, Crilly KS, Kiss Z (1995). Synergistic potentiating effects of choline phosphate and ethanolamine on insulin-induced DNA synthesis in NIH 3T3 fibroblasts. Biochem. Biophys. Res. Commun..

[44] Callery PS, Geelhaar LA (1984). Biosynthesis of 5-aminopentanoic acid and 2-piperidone from cadaverine and 1-piperideine in mouse. J. Neurochem..

[45] Syrjänen S, Piironen P, Markkanen H (1987). Free amino-acid content of wax-stimulated human whole saliva as related to periodontal disease. Arch. Oral Biol..

[46] Kanan, Y. & Al-Ubaidi, M. Tyrosine o sulfation: An overview. *JSM Biotechnol. Biomed. Eng*. **1** (2013).

[47] Kehoe JW, Bertozzi CR (2000). Tyrosine sulfation: A modulator of extracellular protein–protein interactions. Chem. Biol..

[48] Yang Y-S (2015). Tyrosine sulfation as a protein post-translational modification. Molecules.

[49] Bostanci N (2015). Secretome of gingival epithelium in response to subgingival biofilms. Mol. Oral Microbiol..

[50] Bao K, Belibasakis GN, Selevsek N, Grossmann J, Bostanci N (2015). Proteomic profiling of host-biofilm interactions in an oral infection model resembling the periodontal pocket. Sci. Rep..

[51] Mallick S, Benson R, Rath GK (2016). Radiation induced oral mucositis: A review of current literature on prevention and management. Eur. Arch. Otorhinolaryngol..

[52] Brito-Zerón P (2016). Sjögren syndrome. Nat. Rev. Dis. Primers.

[53] Runnel R (2013). Effect of three-year consumption of erythritol, xylitol and sorbitol candies on various plaque and salivary caries-related variables. J. Dent..

[54] Mäkinen KK (2005). Similarity of the effects oferythritol and xylitol on somerisk factors of dental caries. Caries Res..

[55] Honkala S (2014). Effect of erythritol and xylitol on dental caries prevention in children. Caries Res..

[56] Löe H, Theilade E, Jensen SB (1965). Experimental gingivitis in man. J. Periodontol..

[57] World medical association declaration of Helsinki: Ethical principles for medical research involving human subjects (2013). JAMA.

[58] Prodan A (2015). Interindividual variation, correlations, and sex-related differences in the salivary biochemistry of young healthy adults. Eur. J. Oral Sci..

[59] Zwietering MH, Jongenburger I, Rombouts FM, van ‘t Riet K (1990). Modeling of the bacterial growth curve. Appl. Environ. Microbiol..

[60] Fernandez-Gutierrez, M. M., Kleerebezem, M., van Baarlen, P., van Zessen, D. B. H. & Stubbs, A. P. Kreap: An automated galaxy platform to quantify *in vitro* re-epithelialization kinetics. *GigaScience* **7** (2018). 10.1093/gigascience/giy078 PMC6048990 29961849

[61] Pedregosa F (2011). Scikit-learn: Machine learning in python. J. Mach. Learn. Res..

[62] Warnes, G. *et al*. Gplots: Various R programming tools for plotting data. *R J*. (2016).

