# Supplementary material for: A salivary metabolite signature that reflects gingival host-microbe interactions: instability predicts gingivitis susceptibility
Source: Sci Rep. 2020 Feb 20;10:3008. doi: 10.1038/s41598-020-59988-z (PMC7033112; doi:10.1038/s41598-020-59988-z)
Supplement: Supplementary file 1 — Supplementary Information. [file 41598_2020_59988_MOESM1_ESM.pdf]

Supplementary Information

A salivary metabolite signature that reflects gingival host-microbe interactions: instability predicts gingivitis susceptibility

Marcela M. Fernandez-Gutierrez[1,2], Sultan Imangaliyev[1,3,6], Andrei Prodan[1,4,6], Bruno G. Loos[1,5], Bart J. Keijser[1,3,6], Michiel Kleerebezem[1,2*]

[1]TI Food and Nutrition, Nieuwe Kanaal 9-A, 6709 PA, Wageningen, The Netherlands.

[2]Host-Microbe Interactomics Group, Department of Animal Sciences, Wageningen University & Research, De Elst 1, 6708 WD, Wageningen, The Netherlands.

[3] TNO Microbiology and Systems Biology, Utrechtseweg 48, 3704 HE, Zeist, The Netherlands.

[4]Department of Oral Biochemistry, Academic Centre for Dentistry Amsterdam (ACTA), University of Amsterdam and Vrije Universiteit Amsterdam, Gustav Mahlerlaan 3004, 1081 LA, Amsterdam, The Netherlands.

[5]Department of Periodontology Academic Centre for Dentistry Amsterdam (ACTA), University of Amsterdam and Vrije Universiteit Amsterdam, Gustav Mahlerlaan 3004, 1081 LA, Amsterdam, The Netherlands.

[6]Department of Preventive Dentistry, Academic Centre for Dentistry Amsterdam (ACTA), University of Amsterdam and Vrije Universiteit Amsterdam, Gustav Mahlerlaan 3004, 1081 LA, Amsterdam, The Netherlands.

***Correspondence:**
Michiel Kleerebezem
Host-Microbe Interactomics, Wageningen University, De Elst 1, 6708 WD, Wageningen, The Netherlands
Tel: +31 317 483822
Email: michiel.kleerebezem@wur.nl

Supplementary Figures

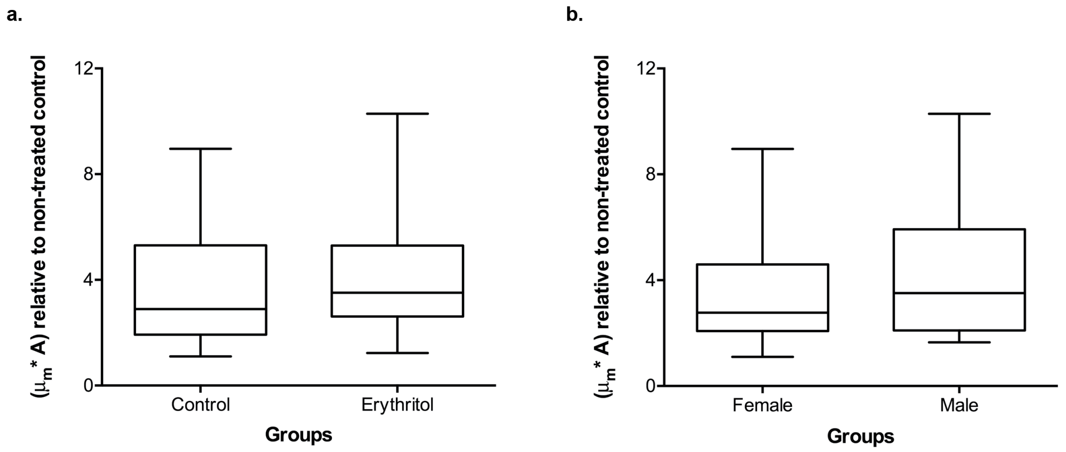

Figure S1. Effect of erythritol and gender on re-epithelialization kinetics. (a) Salivary re-epithelialization capacity was not influenced by daily intake of erythritol (P = 0.44). Control group, n = 72, erythritol group, n = 31. (b) Gender did not influence the capacity of unstimulated saliva to promote *in vitro* re-epithelialization (P = 0.12). Significant differences were assessed by a two-tailed t-test.

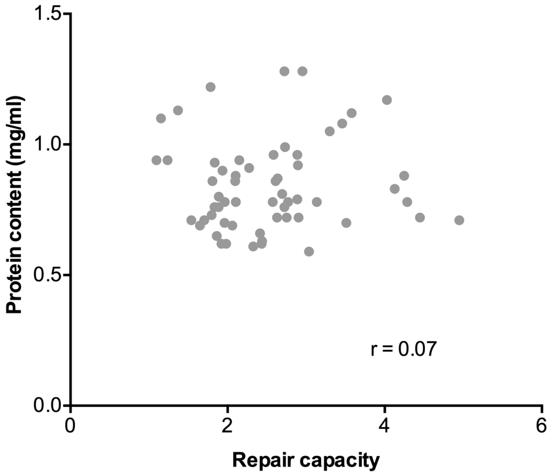

Figure S2. Correlation between unstimulated saliva total protein content and its re-epithelialization capacity. Association between total protein content (mg/ml) and the capacity of unstimulated saliva to promote wound repair on gingival epithelial cells assessed by a Spearman correlation (n = 58, P = 0.58).

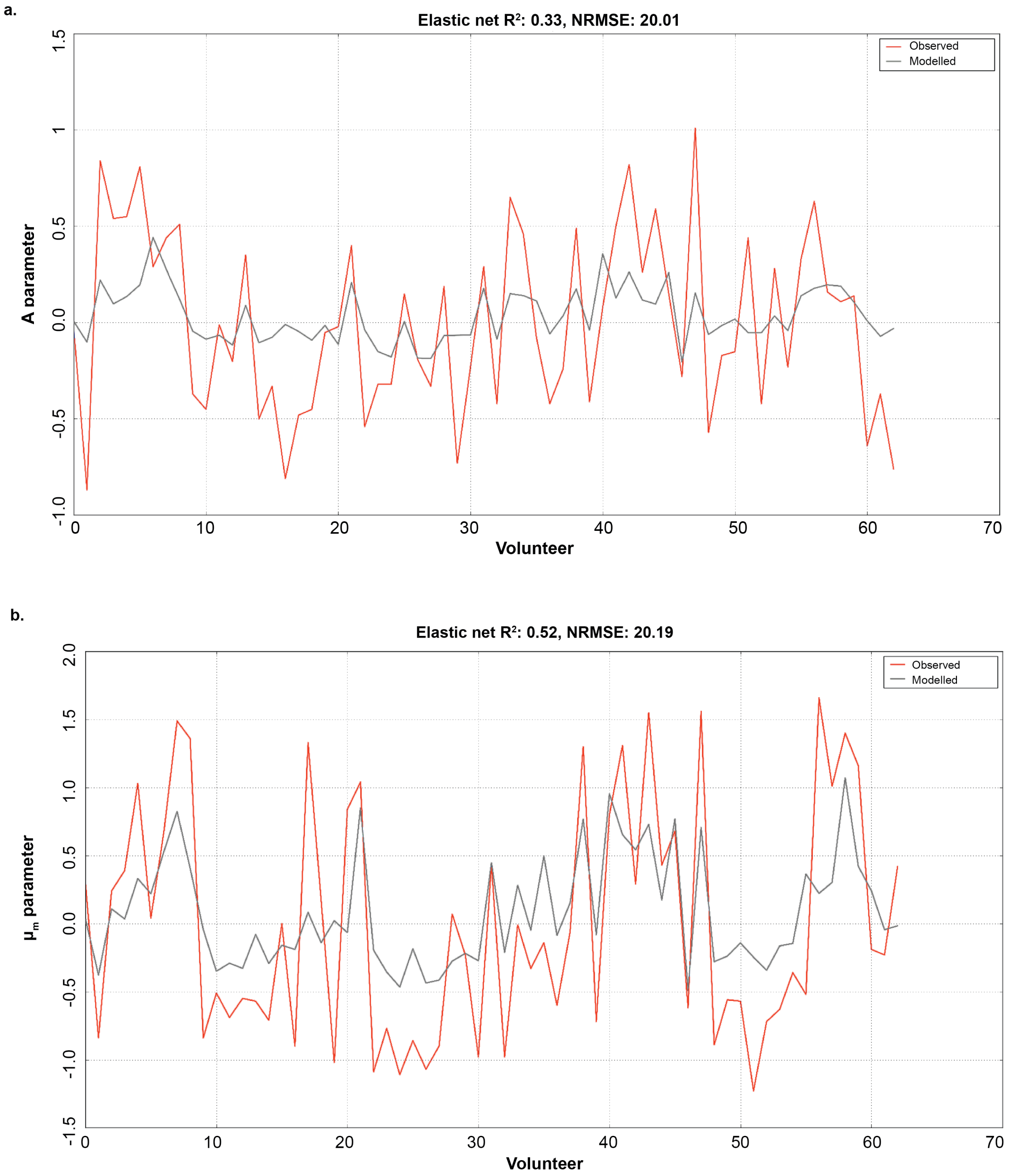

Figure S3. Elastic net regression. (a) Performance of the elastic net regression through the A parameter values. (b) Performance of the elastic net regression through the μ_m parameter values.

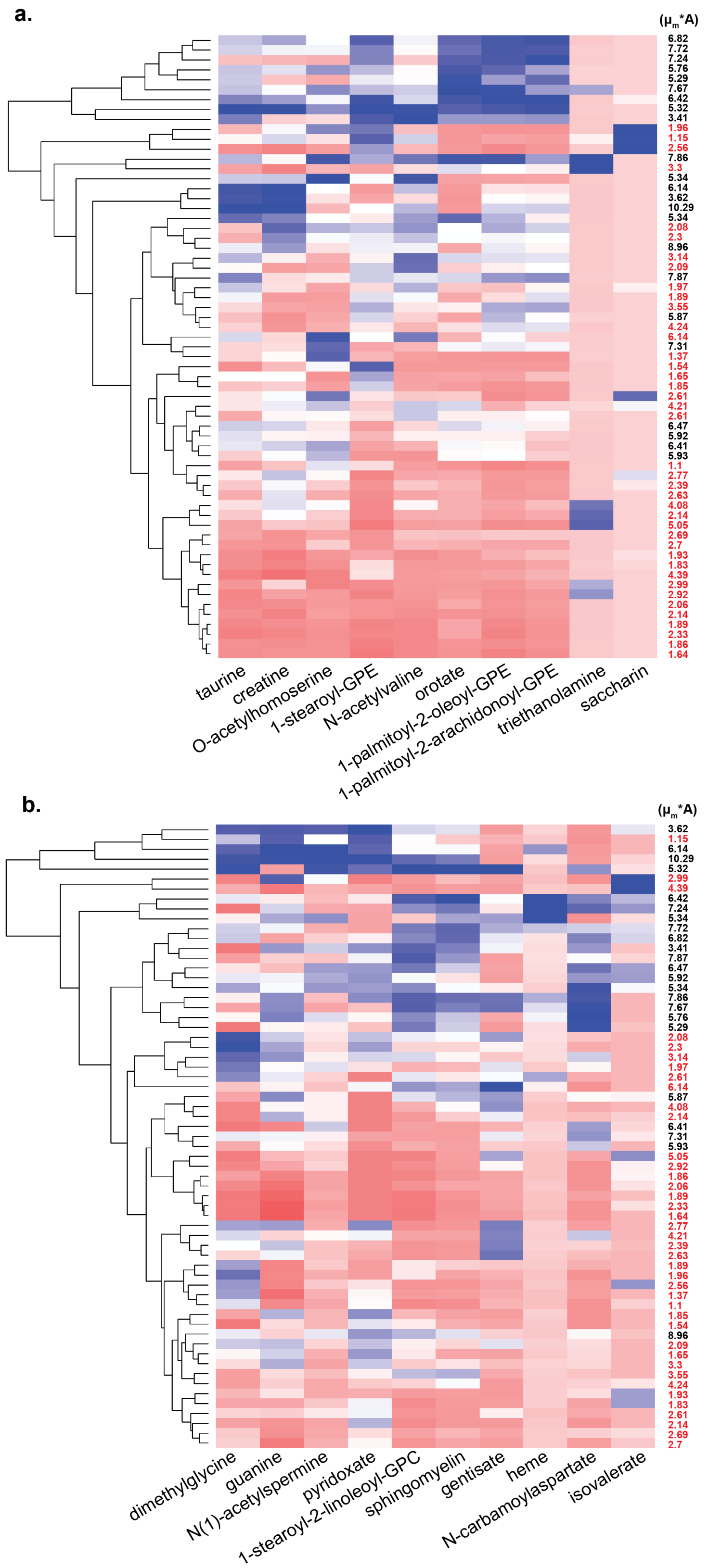

Figure S4. Heatmaps with hierarchical clustering. (a) and (b) are exemplary and representative heatmaps of randomly selected metabolites in the dataset. Re-epithelialization performance values are colour-coded according to their positioning in cluster I (black) or II (red) derived from the hierarchical clustering of the metabolite signature.

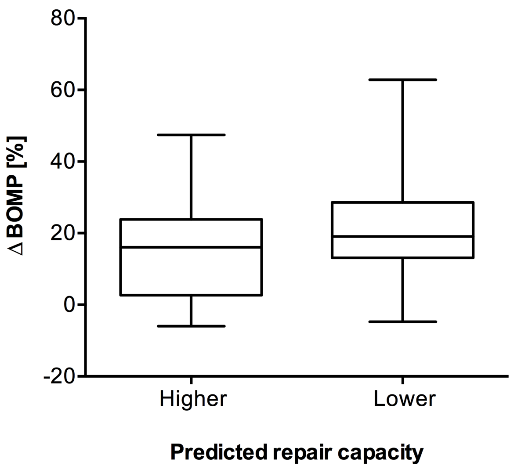

Figure S5. Predictive capacity of the metabolite signature in relation to the response to the challenge. Individuals were split into two groups by the median of the predicted re-epithelialization value (unscaled) at the baseline of the study. The response to the challenge was reflected by the change in the percentage of gingival bleeding in the groups predicted to have saliva samples with higher (n = 30) or lower (n = 31) re-epithelialization capacity. Significant differences were assessed by a two-tailed t-test. No significant difference was found between the groups.

Supplementary Tables

Table S1. Re-epithelialization kinetics obtained for the training dataset. Re-epithelialization kinetic parameters obtained for 63 randomly selected samples collected during the challenge intervention phase (Day 0 to Day 14, timepoint 2 to 6). Samples were tested in duplicates.

Volunteer	Timepoint	Mean, μ_m	SD, μ_m	Mean, A	SD, A	μ_m * A
1	2	1.861	0.075	1.401	0.048	2.607
	3	1.849	0.002	1.422	0.032	2.629
	4	2.727	0.475	1.542	0.163	4.206
	5	2.063	0.745	1.341	0.162	2.767
	6	1.185	0.121	2.015	0.201	2.388
3	2	1.439	0.683	1.34	0.05	1.928
	3	1.908	0.264	1.124	0.161	2.144
	4	2.494	0.502	1.76	0.116	4.39
	5	1.865	0.084	1.375	0.118	2.564
	6	1.707	0.016	1.073	0.124	1.831
5	3	4.077	0.669	2.198	0.248	8.959
8	2	1.403	0.68	1.523	0.202	2.137
	3	3.262	0.356	1.547	0.276	5.047
	4	3.75	1.336	1.087	0.158	4.076
	5	2.593	0.247	1.124	0.047	2.915
	6	2.416	0.298	1.239	0.144	2.994
9	3	2.407	0.467	2.22	0.473	5.343
	4	2.846	0.12	2.157	0.327	6.138
10	3	3.104	0.812	1.719	0.174	5.337
	4	2.714	0.76	2.385	0.238	6.474
	5	3.434	0.684	1.725	0.08	5.925
11	2	3.913	0.593	2.007	0.066	7.855
	3	3.717	0.232	2.064	0.129	7.675
	4	3.102	0.701	1.856	0.093	5.758
15	6	3.218	0.896	1.653	0.078	5.319
16	2	1.703	0.069	1.159	0.172	1.975
	3	1.58	0.226	1.197	0.021	1.891
	4	2.359	0.004	1.329	0.074	3.135
	5	1.822	0.227	1.148	0.054	2.092
	6	2.282	0.223	1.494	0.233	3.408
17	3	2.461	0.537	2.385	0.414	5.869
21	2	1.314	0.365	1.248	0.177	1.639
	3	1.649	0.038	1.249	0.015	2.06
	4	1.803	0.147	1.293	0.023	2.331
	5	1.521	0.029	1.24	0.065	1.886
	6	1.349	0.081	1.38	0.016	1.862
26	3	2.226	0.097	0.934	0.167	2.08
	4	2.84	1.044	0.811	0.147	2.302
	5	2.188	0.404	1.195	0.077	2.615
35	3	2.848	0.606	1.857	0.299	5.289
	4	3.785	0.688	2.079	0.25	7.868
36	2	1.734	0.209	1.557	0.149	2.699
	3	1.564	0.04	1.718	0.189	2.687
	4	1.848	0.217	1.922	0.133	3.552
	5	1.785	0.007	1.849	0.047	3.301
	6	2.09	0.071	2.029	0.097	4.242
45	2	3.727	0.437	2.07	0.301	7.717
	3	3.966	0.216	1.825	0.453	7.239
	5	3.82	1.001	1.68	0.045	6.417
	6	3.463	0.733	1.97	0.131	6.822
51	3	1.905	0.371	1.903	0.692	3.625
	4	3.983	0.07	2.583	0.298	10.289
	6	3.585	0.089	1.714	0.342	6.144
54	4	2.806	0.247	2.112	0.164	5.926
	5	3.453	0.221	2.118	0.19	7.314
	6	2.662	0.385	2.407	0.189	6.408
55	2	1.516	0.073	0.761	0.009	1.153
	3	1.576	0.168	0.701	0.046	1.104
	4	1.329	0.09	1.032	0.025	1.371
	5	1.699	0.174	1.151	0.116	1.956
	6	1.531	0.052	1.003	0.056	1.535
57	3	2.192	0.625	0.842	0.218	1.845
	6	1.437	0.021	1.15	0.206	1.652

Table S2. Feature selection. Elastic net regression with stability selection was performed to select a set of metabolites that were associated to re-epithelialization kinetics using the μ_m and A parameters.

	Feature Selected	Pathway	Stability	Average Weight
**μ_m parameter**	1-(1-enyl-palmitoyl)-2-arachidonoyl-GPE (P-16:0/20:4)*	Plasmalogen	0.60	0.04
	1-(1-enyl-palmitoyl)-2-oleoyl-GPC (P-16:0/18:1)*	Plasmalogen	0.73	0.08
	nicotinate	Nicotinate and Nicotinamide Metabolism	0.67	0.04
	O-sulfo-L-tyrosine	Chemical	1.00	0.15
	urea	Urea cycle; Arginine and Proline Metabolism	0.68	-0.03
**A parameter**	1-stearoyl-GPS (18:0)*	Lysolipid	0.72	-0.03
	2-piperidinone	Food Component/Plant	0.71	0.03
	4-hydroxyphenylacetate	Phenylalanine and Tyrosine Metabolism	0.73	0.03
	glycosyl-N-palmitoyl-sphingosine	Sphingolipid Metabolism	0.76	0.04
	imidazole lactate	Histidine metabolism	1.00	0.07
	phenol sulfate	Phenylalanine and Tyrosine Metabolism	0.81	-0.04

Table S3. Predicted re-epithelialization values and gingival bleeding scores measured during the experimental gingivitis challenge. The coefficient of variation (CV) of the predicted re-epithelialization kinetics was defined as the ratio of the standard deviation to the mean * 100. Percentage of bleeding on marginal probing (BOMP%) was measured at the baseline (Day 0) and peak of the challenge (Day 14).

Volunteer #	Mean (μ_m*A)	SD (μ_m * A)	CV (%)	BOMP% (Day 0)	BOMP% (Day 14)	Δ BOMP%
1	1.86	0.18	9.82	3.57	17.86	14.29
2	1.42	0.14	9.78	17.86	13.1	-4.76
3	1.54	0.12	7.53	5.95	27.38	21.43
4	7.36	3.43	46.64	15.38	50	34.62
5	2.34	0.73	31.32	3.85	66.67	62.82
6	3.46	0.36	10.32	9.52	3.57	-5.95
7	1.60	0.29	18.23	10.71	11.9	1.19
8	1.95	0.26	13.24	16.67	38.1	21.43
9	3.01	0.74	24.78	3.57	34.52	30.95
10	3.57	0.56	15.70	4.76	26.19	21.43
11	3.76	0.78	20.71	9.52	10.71	1.19
12	1.82	0.35	19.09	6.94	30.95	24.01
13	2.83	0.26	9.30	4.76	35.71	30.95
14	1.89	0.32	16.82	8.33	44.05	35.72
15	4.04	1.26	31.23	16.67	46.43	29.76
16	2.59	0.70	27.09	5.13	52.56	47.43
17	2.63	0.58	21.99	2.38	9.52	7.14
18	2.21	0.45	20.52	2.38	19.05	16.67
19	2.43	0.12	4.97	9.52	33.33	23.81
20	1.61	0.17	10.43	6.41	23.08	16.67
21	1.16	0.14	12.43	1.19	16.67	15.48
22	1.45	0.22	15.25	1.19	4.76	3.57
23	2.65	0.45	17.08	10.71	21.43	10.72
24	2.08	0.49	23.53	2.38	17.86	15.48
25	2.33	0.53	22.75	1.28	30.77	29.49
26	2.34	0.30	12.93	19.23	34.62	15.39
27	2.94	0.36	12.13	10.71	30.95	20.24
28	1.71	0.22	12.93	0	29.76	29.76
29	3.12	0.43	13.81	7.14	7.14	0
30	1.31	0.14	10.58	1.19	10.71	9.52
31	1.65	0.10	6.22	9.72	26.39	16.67
32	1.20	0.09	7.80	1.19	8.33	7.14
33	1.44	0.19	12.97	16.67	30.95	14.28
34	2.03	0.23	11.47	2.38	17.86	15.48
35	2.81	0.87	31.01	2.78	5.56	2.78
36	1.98	0.26	13.38	10.71	21.43	10.72
37	1.95	0.55	28.29	4.76	36.9	32.14
38	3.09	0.74	23.96	0	2.38	2.38
39	2.67	0.75	28.23	8.33	33.33	25
40	1.14	0.06	5.11	4.76	13.1	8.34
41	1.32	0.15	11.21	2.38	23.81	21.43
42	2.50	0.28	11.11	5.95	22.62	16.67
43	1.39	0.12	8.94	3.57	20.24	16.67
44	2.54	0.12	4.81	5.13	25.64	20.51
45	4.33	0.35	8.06	22.22	18.06	-4.16
46	1.24	0.22	18.02	11.11	33.33	22.22
47	1.58	0.17	10.50	16.67	42.31	25.64
48	1.98	0.17	8.38	0	5.95	5.95
49	1.29	0.14	10.70	8.33	21.43	13.1
50	2.36	0.38	15.94	13.1	41.67	28.57
51	3.53	0.97	27.48	19.05	45.24	26.19

52	3.36	0.56	16.78	3.57	32.14	28.57
53	1.46	0.29	19.90	3.57	22.62	19.05
54	3.23	0.45	14.01	4.76	5.95	1.19
55	1.84	0.29	15.72	2.38	11.9	9.52
56	1.94	0.49	25.07	17.86	45.24	27.38
57	1.64	0.20	12.49	2.78	9.72	6.94
58	4.52	1.17	25.89	22.62	45.24	22.62
59	3.31	0.37	11.21	9.52	25	15.48
60	1.44	0.14	9.59	4.76	34.52	29.76
61	1.59	0.31	19.81	1.19	28.57	27.38
